# Transcriptomic Meta-Analysis Identifies Upregulated Clotting and Fibrinolysis Pathways in Colorectal Cancer Tumors Containing Hereditary PMS2 Mismatch Repair Deficiency

**DOI:** 10.17912/micropub.biology.001159

**Published:** 2024-07-27

**Authors:** Trenton M Gibson, Mauri D Spendlove, Naomi Rapier-Sharman, Brett E Pickett

**Affiliations:** 1 Microbiology and Molecular Biology, Brigham Young University, Provo, Utah, United States; 2 Microbiology and Molecular Biology, Brigham Young University

## Abstract

Lynch Syndrome is characterized by deficient mismatch repair (dMMR) components. We performed a meta-analysis of multiple RNA-sequencing datasets from patients with different dMMR variants (PMS2, MLH1, and MSH2) to better characterize the unique transcriptional profiles. Our results reveal enriched signaling pathways from tumor samples with germline mutations in the PMS2 gene including upregulation in pathways related to intrinsic and extrinsic prothrombin activation, fibrinolysis, and uPA/uPAR-mediated signaling. These pathways have been associated with tumor growth, invasiveness, and metastasis. This work provides support for further exploration into the role of PMS2 in tumor development, and as a potential therapeutic mechanism.

**Figure 1.**
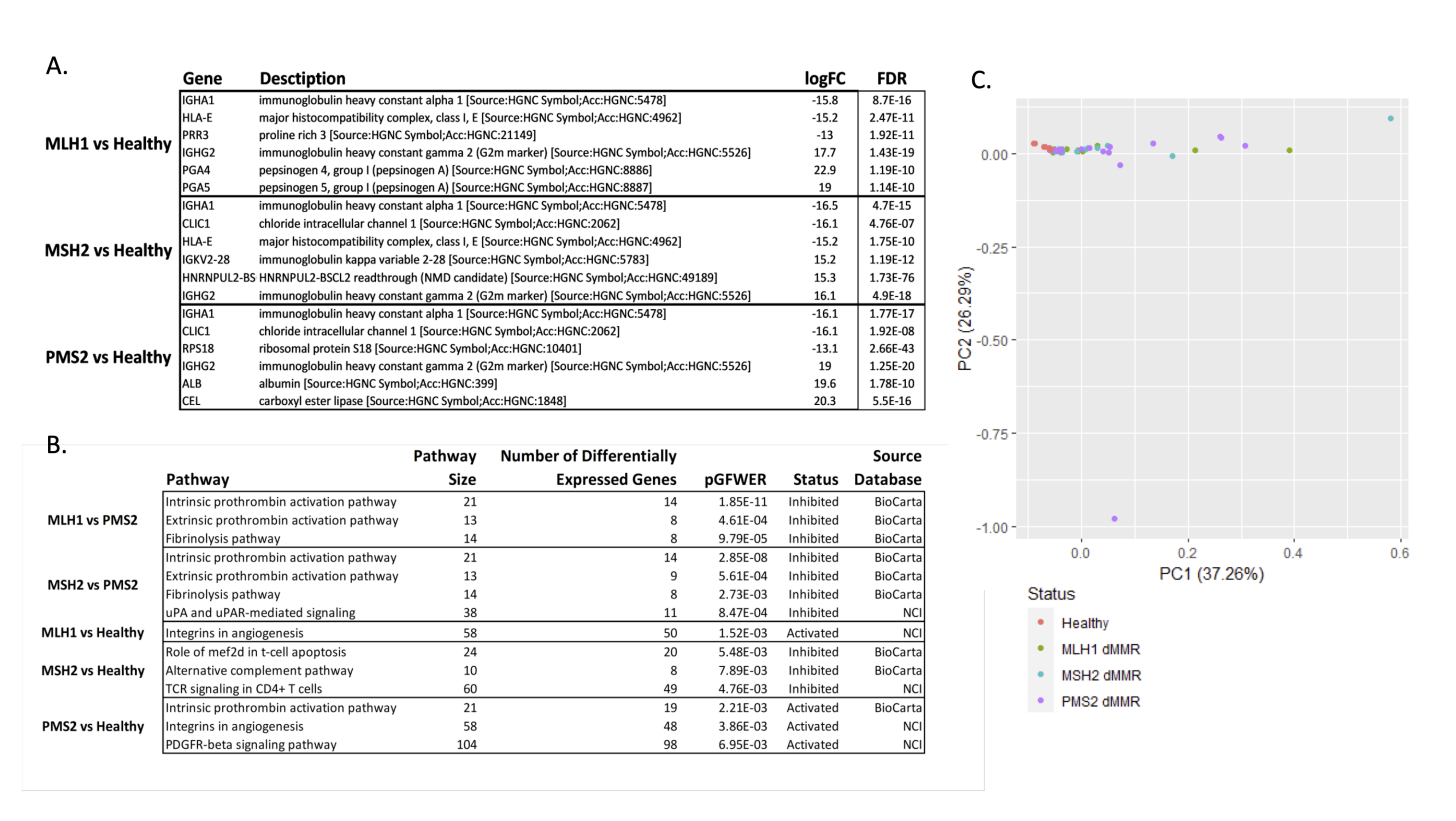
**A.** Table of Differentially Expressed Genes in Lynch Syndrome Tumors. Depicts the top three upregulated and top three downregulated genes for various Lynch Syndrome dMMR variants (in the MLH1, MSH2, or PMS2 genes) compared to healthy colon samples. **B.** Table of Most Significant Signaling Pathway Impact Analysis (SPIA) Results . Multiple comparisons highlight the activated pathways in tumor samples vs. healthy control samples, characterized by PMS2 dMMR compared to MLH1 and MSH2 deficient patients. **C.** Principal Component Analysis (PCA) of Gene Expression Levels. A PCA plot indicating the relationships among RNA sequencing samples obtained from GEO. Samples were color-coded by categories including controls and dMMR germline mutations. Healthy, non-cancerous colorectal epithelial samples form a noticeable cluster, while patients with different MMR mutations exhibit heterogeneity in gene expression.

## Description

Lynch Syndrome is an autosomal dominant disorder in which the endogenous DNA mismatch repair (MMR) system becomes deficient (dMMR). Deleterious germline mutations in any gene product within the system ( *MLH1, MSH2, MSH6,* and *PMS2* ) severely reduce the ability of the cell to find and repair spontaneous variants that occur across the somatic genome (1) , (2) . The protein products of these genes dimerize to form many of the components in the MMR protein complex. For example, MSH2 dimerizes with MSH6 to form the MutSα subunit and MLH1 dimerizes with PMS2 to form the MutLα subunit (3) .

Individuals with dMMR have a higher prevalence of genomic instability since the inability to repair spontaneous variants increases the frequency of DNA nucleotide mismatches. These underlying issues cause increases in the occurrence of homologous recombination (4–6) and of developing cancer (7) . Genomic stability in cancer patients is quantified by determining the relative abundance of microsatellite repeats in tumor cells compared to healthy somatic cells. Microsatellite instability (MSI) is a classification status given to tumors with differing numbers of microsatellite repeats than other somatic cells and is a defining characteristic of Lynch Syndrome (8) .

The aim of this study was to identify differentially expressed genes and enriched signaling pathways among cancer tumors of patients with germline mutations in the relevant MMR genes. Particularly, we performed a transcriptomic meta-analysis to compare the expression from Lynch Syndrome samples where mutations were present in one of three dMMR genes both to each other and to non-cancerous colon samples. This study design was required since one study only contained samples from Lynch Syndrome patients who had mutations in dMMR genes, while a separate second study contained the healthy control samples.

We performed separate differential expression analyses of genes in the patients with mutations in the MLH1, MSH2, or PMS2 genes, compared to healthy controls. Differential expression analyses were also performed between the different dMMR variants (e.g. MLH1 vs PMS2, MLH1 vs MSH2, and MSH2 vs PMS2 variants). Comparative analyses including MSH6 were not performed because of the lack of a sufficient sample size to yield significant results. We focused our analysis on the six genes with the biggest log _2_ fold-change values for each contrast ( Figure 1A ). Interestingly, when we compared these results, we noticed that the IGHA1, IGHG2, and HLA-E genes were differentially expressed across two or more comparisons.

We then used the DEGs from each comparison to calculate the intracellular signaling pathways that were most significantly affected using the signaling pathway impact analysis (SPIA) algorithm. We observed that tumor samples characterized by PMS2 dMMR contain multiple inhibited pathways when compared to MLH1- and MSH2-deficient patients ( Figure 1B ). When comparing MLH1 dMMR to PMS2 dMMR, we found that the intrinsic and extrinsic prothrombin activation pathways were inhibited as well as the fibrinolysis pathway. The contrast of MSH2 dMMR with PMS2 dMMR showed these same pathways being inhibited as well as the uPA and uPAR-signaling pathway. These findings suggest a possible effect of PMS2 on proteins in the prothrombin and fibrinolysis pathways (9–12) . In fact, other studies have shown that these pathways are often upregulated in cancer and play a role in tumor growth, angiogenesis, and metastasis. Specifically, fibrin degradation products (FDP), small protein fragments resulting from the breakdown of fibrin clots, have been shown to promote malignancy in cancer cells (13–16) . Interestingly, our contrast between MLH1 and MSH2 did not reveal any significant pathways. We next performed a principal component analysis (PCA) to gain a better understanding of the transcriptional relationships between the samples ( Figure 1C ). This analysis showed that healthy, non-cancerous colorectal epithelial samples were the only group that showed any noticeable clustering, suggesting at least some similarities in the gene expression profiles among the dMMR samples. Another potential interpretation for this lack of clustering among the patients with different MMR mutations is that it accurately reflects the wide heterogeneity of gene expression that is common in cancerous cells.

We analyzed the top DEGs from the cancer vs healthy control comparisons in the University of Alabama at Birmingham Cancer data analysis portal (UALCAN) to better understand the clinical importance of our findings (17) . We focused this analysis on the PMS2 vs healthy control comparison since it had some of the most statistically significant DEGs in our initial results. In particular, we found that the carboxyl ester lipase (CEL) gene product was significantly upregulated across all colorectal cancer stages, as well as at significantly higher transcriptional levels both before and after lymph node metastasis. The CEL gene product was also detected at higher levels in the CPTAC proteomics data (18) . We observed that the CEL gene locus had a significantly broader range of methylation in tumor samples than in healthy controls, and that higher expression of this gene led to a non-significant increase in patient survival. It is difficult to filter for colorectal cancer samples in these platforms that were caused directly by MMR mutations. A similar analysis using the PanCancer Atlas data in cBioPortal revealed that missense mutations in the PMS2 gene was associated with a much broader range of CEL expression (19) . Consequently, additional validation experiments are justified, particularly given the relatively small numbers of public samples that could be included in this analysis.

We then tried to identify existing therapeutics that could be repurposed for Lynch Syndrome using Pathways2Targets; however, there were no known targets in those significant signaling pathways. This result is somewhat expected given the relatively low number of enriched signaling pathways and the lack of drugs targeting those pathways. However, this negative result suggests that future work towards the development of drugs targeting proteins in the prothrombin and/or fibrinolysis pathways as potential therapeutics for colorectal cancer and Lynch syndrome could be beneficial. A potential limitation of this study is the inability to confirm the diversity of genetic backgrounds represented in the patient populations, which should be addressed in future studies. In the emerging era of personalized medicine, our results could support the exploration and development of therapeutics targeting these pathways only for individuals with mutations in the PMS2 locus.

## Methods

The RNA-sequencing datasets were found on the National Center for Biotechnology Information (NCBI) Gene Expression Omnibus (GEO) database. The search term “Lynch syndrome” was used and only human RNA-sequencing datasets were selected for screening. Of the 11 results available as of January 17th, 2024; only one study met our filtering criteria (GSE146889). Specifically, these criteria involved including only case samples that characterized the specific dMMR variants (MLH1, MSH2, MSH2, and PMS2) in Lynch Syndrome patients in our analyses. The other samples were excluded for various reasons that included: data collected from cell lines, organoids, or patients in interventional clinical trials and were not conducive to the purpose of our study. Fifty-five of these publicly available bulk RNA-sequencing files were obtained from patients with colorectal cancer and high microsatellite instability. A separate study containing 20 non-cancerous colon tissue samples was used as a healthy control (GSE214695). Cluster visualization using principal component analysis was performed in R.

Fastq files containing the RNA-sequencing read data were downloaded from the Sequence Read Archive (SRA) using the sra-tools software. The Fastq files, the associated metadata (Supplementary Table S1), and a configuration file were used as inputs to the Automated Reproducible MOdular workflow for preprocessing and differential analysis of RNA-seq data (ARMOR) workflow (20) . The ARMOR workflow involved quality control, removing sequencing adapters and low-quality regions with TrimGalore! , read pseudomapping and quantification to the human GRCh38 transcriptome using Salmon (21) , calculation of significant differentially expressed genes using edgeR (22) , and gene ontology enrichment analysis using Camera (23) .

The differentially expressed genes (Lynch Syndrome vs healthy colorectal tissue) generated by the ARMOR software were categorized as statistically significant if the false discovery rate adjusted p-value < 0.05. These significant genes became the input for the signaling pathway impact analysis (SPIA) software, which identified significantly enriched signaling pathways (Bonferroni-adjusted p-values < 0.05) from the following databases: KEGG, Panther, BioCarta, Reactome, and NCI (24–28) The output of SPIA became the input for a drug repurposing and target prioritization algorithm, Pathways2Targets (29) , which uses more than 20 attributes from the OpenTargets database and pathway information to rank and prioritize therapeutic targets based on customizable criteria.
